# Dietary methionine supplementation to a low-protein diet improved hair follicle development of Angora rabbits

**DOI:** 10.5713/ab.22.0286

**Published:** 2022-11-14

**Authors:** Man Zhao, Tongtong Wang, Bin Wang, Chuanhua Liu, Fuchang Li, Lei Liu

**Affiliations:** 1 Shandong Provincial Key Laboratory of Animal Biotechnology and Disease Control and Prevention, Department of Animal Science, Shandong Agricultural University, Taian, Shandong 271018, China; 2 Animal Husbandry Development and Promotion Center of Mengyin County, Linyi, Shandong 276200, China

**Keywords:** Hair Follicle Growth, Low Protein Diet, Methionine, Nitrogen Metabolism

## Abstract

**Objective:**

Angora rabbits fed a low-protein diet exhibit decreased hair production performance. This study was set out to evaluate the effects of methionine on hair properties and nitrogen metabolism in Angora rabbits fed a low-protein diet and to investigate the gene expression related to hair follicle development to determine the possible molecular mechanism of methionine effects on hair follicle development.

**Methods:**

An experiment was conducted to investigate the effects of DL-methionine addition on a low-protein diet on hair development in Angora rabbits. Angora rabbits were divided into 5 groups: fed a normal diet (control), fed a low-protein diet (LP), or fed an LP supplemented with 0.2%, 0.4%, or 0.6% DL-methionine (Met).

**Results:**

The results showed that rabbits in the LP group had lower wool yield than the control rabbits, but the addition of 0.4% to 0.6% Met to LP attenuated these effects (p< 0.05). Dietary addition of 0.4% to 0.6% Met to LP increased the apparent nitrogen digestibility, nitrogen utilization rate, and feed efficiency (p<0.05). Feeding LP decreased the insulin-like growth factor 1 (*IGF1*), keratin-associated protein (*KAP*) 3.1, and *KAP* 6.1 mRNA levels compared with the control, but the addition of 0.4% Met in LP attenuated these effects (p<0.05). Relative to the LP or control group, dietary addition of 0.4% Met increased versican mRNA levels.

**Conclusion:**

In conclusion, the addition of Met to LP could improves wool production performance and feed efficiency and reduce nitrogen emissions in Angora rabbits. Met can promote hair follicle development, which may be associated with *IGF1*, *KAP*, and the versican signaling.

## INTRODUCTION

Many studies have shown that hair growth and hair properties are very dependent on the dietary protein supply [1]. Low dietary protein levels may not be able to maintain the wool production demand of fur animals, thus reducing the wool production efficiency [2]. As the most limiting amino acid (AA), methionine (Met) plays an important role in fur animals. Met can be converted into S-adenosylmethionine (SAM), after which it can undergo hydrolysis to produce homocysteine [3]. Protein synthesis in animal hair requires homocysteine. Recent literature has shown that Met can increase the density of hair follicles on the back skin *in vitro* and prolong hair stem growth *in vivo* [4].

Angora rabbits are important small herbivores that produce wool. The quality of Angora rabbit fur depends on the density of rabbit hair. Previous literature shows that hair follicle density can determine hair density [5]. The hair follicle is a complex tissue composed of multiple layers of different types of cells [6]. The growth of hair follicles has the characteristics of a periodic cycle, which repeatedly goes through a rapid proliferation period (Anagen), an apoptosis-regulated degeneration period (Catagen), and a period of stable resting (Telogen) [7]. These cycles continue repetitively throughout a rabbit’s life. The induction of hair follicles, the maintenance of hair shaft growth, and the differentiation of undifferentiated cells in the hair shaft are regulated by specific genes, such as bone *Wnt10b*, versican, hepatocyte growth factor (*HGF*), insulin-like growth factor 1 (*IGF1*), keratin-associated protein (*KAP*), epidermal growth factor (*EGF*), transforming growth factor β (*TGFβ*) and noggin [8]. *Wnt10b*, versican, *HGF*, *IGF1*, *KAP*, *EGF*, and noggin positively regulate hair follicle growth, but *TGFβ* has a negative effect on hair follicle growth. However, scientific information and experimental dates on the effects of proteins and methionine on the expression of genes related to hair follicle growth in rabbits are scarce. It is speculated that methionine may affect wool yield in Angora rabbits by mediating genes related to hair follicle development. In our present study, we evaluated the effects of methionine on hair production performance and nitrogen metabolism in Angora rabbits fed a low-protein diet and investigated the gene expression related to hair follicle development to determine the possible molecular mechanism of how methionine affects hair follicle development.

## MATERIALS AND METHODS

### Animals, experimental design, and management

Angora rabbits (10 months old, n = 225) were individually raised in wire-cages (60 cm×50 cm×50 cm). During the experiment, the temperature and photoperiod were maintained according to commercial conditions (20°C to 23°C, 12:12 L:D). Regular cleaning of rabbit hutches for manure was implemented. All tested Angora rabbits were from the “Tu wang” long-haired rabbit breeding base in Mengyin County, Linyi City, Shandong Province, China. The basal diet was formulated according to rabbit nutritional requirements [9]. The raw material composition and nutrition levels of the basic diets are shown in Table 1. All rabbits had free access to feed and water during the experimental period. All study procedures were approved by the Shandong Agricultural University Animal Care and Use Committee (SDAUA-2019-066) and were in accordance with the Guidelines for Experimental Animals of the Ministry of Science and Technology (Beijing, China). All rabbits (n = 225) with similar body weight (4,638.6±61.54 g) and wool production and with clipped hair were randomly divided into 5 groups according to sex and body weight (45 rabbits per group, 5 replicates per group, 9 rabbits per replicate). Each group was characterized by a unique diet, which included the normal protein diet (control, a diet containing 17.09% CP) and the low protein diet (LP, 15.13% to 15.18% CP). The normal protein diet was supplemented with 0.1% DL-methionine (Met), while the low protein diet was supplemented with 0.1%, 0.2%, 0.4%, or 0.6% Met, with a total Met content of 0.33%, 0.31%, 0.42%, 0.62%, or 0.81% for each of the five groups, respectively. Met was obtained from the Shandong Jiashi Animal Husbandry. The experiment lasted for 70 days, which included a 5-day adaptation period and a 65-day experimental period. Feed intake was recorded daily.

### Sample collection

At the end of the experiment, 10 rabbits in each group (the male-female ratio was 1:1) were selected for metabolic testing for 7 days. 50 rabbits were transferred to each of 50 metabolic cages (60 cm×50 cm×50 cm) for digestion and metabolism experiments and continued feeding. During the metabolic experiment, the corresponding experimental diets were fed, and the rabbits were free to eat and drink. The pre-test period of the digestion and metabolism test was 4 days, and the formal feeding period was 3 days. The wool yield and quality were measured in 175 other rabbits. At the same time, skin samples (n = 10) were collected from each group (male: female ratio was 1:1) for hair follicle density measurement and hair follicle development-related gene analysis, and all efforts were made to minimize suffering.

### Wool production and quality determination

The whole body of the Angora rabbit was shaved with a razor, and the shaved rabbits were weighed (n = 175; 5 replicates per group, 7 rabbits per replicate). 10 Angora rabbits (the male-female ratio was 1:1) were randomly selected from each group, and the average wool yield was calculated for all hair shaved off each experimental rabbit, and the rabbit hairs from the shoulder, back, hip and abdomen were collected. The lengths of the hair from the shoulder, back, hip and abdomen were measured with a ruler, and the average length was calculated. The average length of rabbit hair was measured by the method described in ASTM test method D1234 (ASTM, 2002a). This includes placing staples on a flat plate covered with a black blanket and using a ruler to measure the length of the nearest 1mm from the tip to the base. The coarse wool rate and average wool fineness were measured using an electronic fibre fineness detector (model PS-W-4; Shanghai Juhong Instrument Equipment Co., Ltd. Shanghai, China).


$$\begin{array}{l} \text{Average daily feed intake }(\text{ADFI})\;(\text{g})\\ =\text{Total feed intake of each rabbit during the feeding experiment}/\text{Test days}\\ \text{Feed conversion ratio }(\text{FCR})\\ =\text{Total feed intake of each rabbit during the feeding experiment}/\text{Wool yield of each rabbit during the feeding experiment}. \end{array}$$

### Nitrogen metabolism determination

Feed samples for each group were collected from the feed bags, sealed in plastic bags and stored in an airtight environment at 4°C. At the end of the pre-test period, rabbit faecal samples were collected and weighed for 3 days. Similarly, urine samples were collected to record the urine volume. During the metabolic test, the food intake, faecal discharge, and urine volume of each rabbit were recorded. The collected samples were stored in an airtight environment at a temperature of 4°C. The detailed material for the determination of the chemical composition of the diets and nitrogen metabolism is given in Supplementary Material S1.

### Hair follicle density determination

For anesthesia, 0.7% sodium pentobarbital (6 mL/kg) was injected intravenously into the ear, and the muscle relaxant in the anesthetic did not inhibit respiration. Under anesthesia, skin biopsies (n = 10; the male: female ratio was 1:1) with sizes of approximately 1 cm×1 cm were collected from the back and buttocks of the rabbits, and the shaved rabbit hair was fixed in 4% paraformaldehyde solution. The solution was changed with fresh 4% paraformaldehyde every 7 days, and the samples were stored in paraformaldehyde at 4°C. Skin samples were observed by histological examination and staining. In short, standard paraffin embedding procedures were performed on skin specimens, and sections with a thickness of 5 μm were stained with hematoxylin and eosin (Sigma–Aldrich, St. Louis, MO, USA). Sections were examined under an Olympus CX-41 phase-contrast microscope (Olympus, Tokyo, Japan), and the density of hair follicles was calculated. Detailed materials for paraffin sectioning and staining were given in Supplementary Material S2.

### The expression of genes related to hair follicle development determination

Skin biopsies (n = 10; the male: female ratio was 1:1) with sizes of approximately 1 cm×1 cm were collected from the back and buttocks. According to a previously published method [10], hair follicle cells were isolated from skin samples, frozen in liquid nitrogen, and stored at −80°C.

Total RNA extraction and quantitative real-time polymerase chain reaction (PCR) were performed as described previously [11]. The quality of RNA after DNase treatment was tested by electrophoresis on an agarose gel, and the quantity of RNA was determined using a biophotometer (Eppendorf, Hamburg, Germany). Primer sequences are shown in Table 2. The PCR data were analysed with the 2^−ΔΔCT^ method. The mRNA levels of target genes were normalized to glyceraldehyde 3-phosphate dehydrogenase (*GAPDH*) mRNA (ΔCT). Detailed materials for total RNA extraction and real-time quantitative PCR are given in Supplementary Materials S3. Based on the cycle threshold (CT) value, the *GAPDH* mRNA level was stable across the treatments in this study (p>0.1) (Supplementary Figure S1).

### Statistical analysis

All the data were analyzed with one-way analysis of variance mathematical model Y_ij_ = μ+α_i_+e_j_ (Y, result; μ, intercept; α, the diet effect; e, random error; i = control, LP, LP+0.2% Met, LP+0.4% Met, LP+0.6% Met; j = 1, 2, 3, 4, 5, 6, 7, 8, 9, 10) by using the Statistical Analysis Software (SAS version 8e; SAS Institute, Cary, NC, USA) to estimate the main effect of age. Comparisons between means used Duncan’s Multiple Range test. The threshold for statistical significance was p<0.05. Data are presented as means±standard error.

## RESULTS

### Wool production and quality

Compared with the control, rabbits in the LP group had a low wool yield (Table 3, p<0.05). While 0.2% Met addition did not significantly affect the wool yield compared with the LP group (p>0.05), 0.4% and 0.6% Met addition significantly increased wool yield (p<0.05). Feeding LP did not significantly change the FCR compared with the control (p>0.05). Compared with the LP group, 0.4% and 0.6% Met addition significantly decreased the FCR (p<0.05). Compared with the control, feeding the LP diet significantly decreased the coarse wool rate and wool fineness (p<0.05). With the addition of dietary Met, the inhibitory effect of LP on the coarse wool rate and wool fineness was reversed (p<0.05). LP or Met treatment did not significantly affect wool length (p> 0.05).

### Hair follicle density

Compared with the control, LP or Met treatment did not significantly affect the total follicle density or secondary follicle density (Table 4, p>0.05). Primary follicle density was significantly decreased after feeding the LP diet compared with the control (p<0.05), but primary follicle density significantly increased with Met addition in LP diet (p<0.05).

### Nitrogen metabolism

Compared with the control, LP or LP+Met significantly decreased the intake of nitrogen, faecal nitrogen, and urinary nitrogen (Table 5, p<0.05). LP or Met treatment did not significantly change nitrogen retention (p>0.05). The addition of Met in LP diet significantly increased the apparent nitrogen digestibility, nitrogen utilization rate, and nitrogen biological value (p<0.05).

### Gene expression of hair follicle development

**Figure 1:**
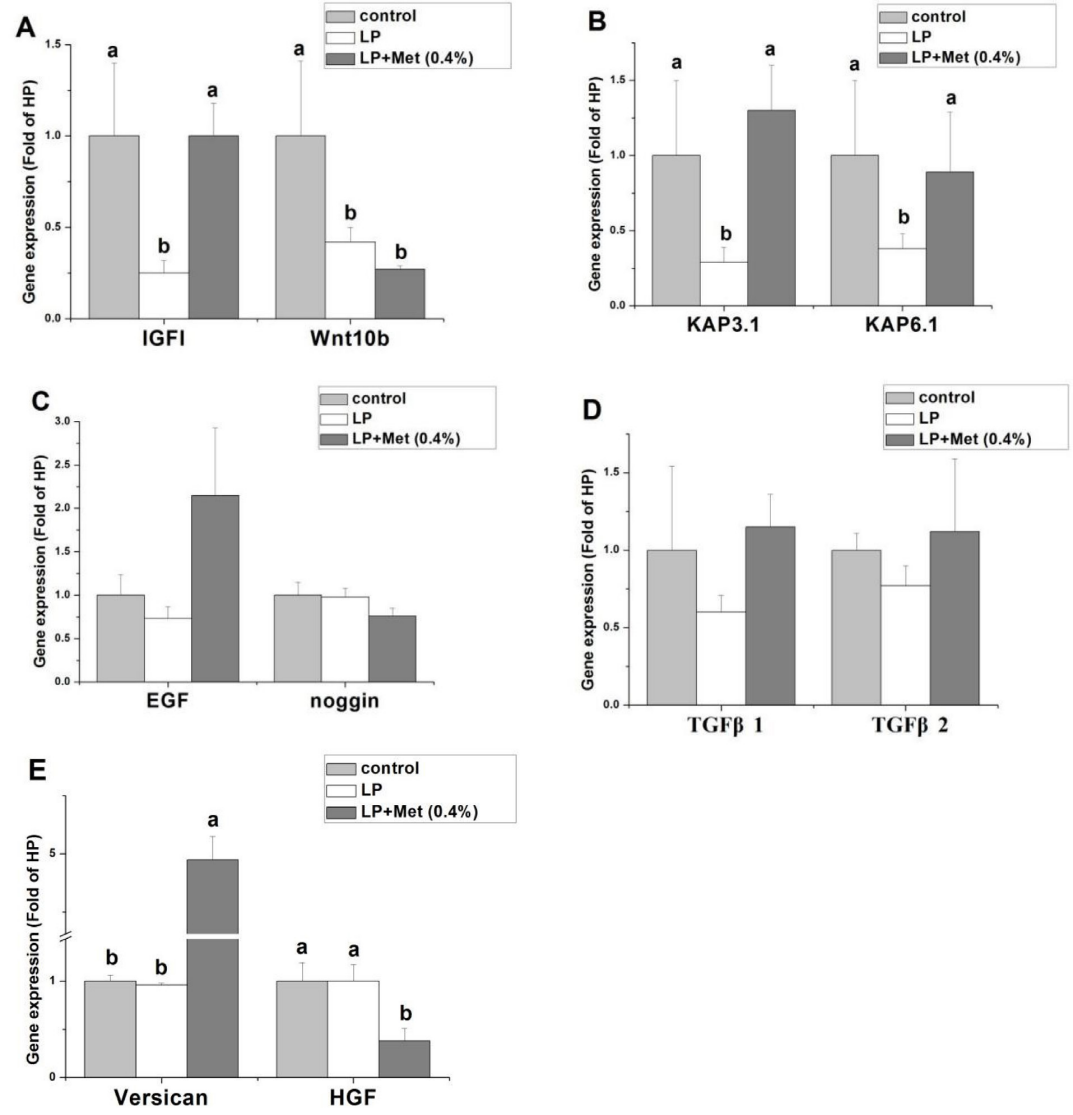
Effect of a low-protein diet and a low-protein diet supplemented with 0.4% methionine on gene expression related to hair follicle development in Angora rabbits (n = 10). Data are presented as means±standard error. IGF1, insulin-like growth factor 1; Wnt10b, Wingless-type MMTV integration site family, member 10B; KAP3.1, Keratin Association Protein 3.1; KAP6.1, Keratin Association Protein 6.1; EGF, epidermal growth factor; TGFβ1, transforming growth factor β1; TGFβ2, transforming growth factor β2; HGF, hepatocyte growth factor. Treatments: control, normal diet; LP, low protein diet; LP+0.4% Met, LP+0.4% methionine. ^a,b^ Values with different superscripts differ significantly (p<0.05).

In this experiment, LP+0.4% Met group had the best performance in wool yield and primary follicle density than other groups and LP+0.6% Met group performed better, so control, LP, and LP+0.4% Met groups were chosen to measure the gene expression related to follicle development. As shown in Figure 1, compared with the control, LP treatment significantly decreased the mRNA levels of *IGF1*, *KAP3.1*, and *KAP6.1* (p<0.05), but dietary Met addition significantly reversed the effects of LP (p<0.05). Compared with the control, LP treatment and LP+0.4% Met treatment significantly decreased the wnt10b mRNA level (p<0.05). Compared with the control, LP treatment and LP+0.4% Met treatment did not significantly alter the gene expression of *EGF*, Noggin, *TGFβ1*, and *TGFβ2* (p>0.05). LP+0.4% Met significantly increased versican gene expression and decreased *HGF* gene expression compared to the control and LP groups (p<0.05).

## DISCUSSION

### Dietary addition of Met improved wool production in Angora rabbits fed a low-protein diet

Adequate high-quality protein and Met are necessary for hair growth. However, the research on methionine in Angora rabbit feed is limited. Mink-fed LP resulted in poor fur quality. The addition of 0.8% Met to LP significantly improved the fur quality and skin weight [2]. After intraperitoneal administration of Met in Angora goats, their wool yield percentage increased by 5.3% compared with the control group [12]. Our data are consistent with these findings, as low protein decreased wool production compared with the control, and dietary addition of 0.4% to 0.6% Met in LP increased wool production to the control level.

The diameter of hair fibre is an important parameter in the textile processing process, which is affected by the dietary Met level. Dietary Met supplementation significantly increased the fiber diameter and improved the wool quality of Angora rabbits [13]. The addition of Met to LP increases the down diameter and improves the fur quality of blue foxes [14]. Our data are consistent with these findings, as feeding LP decreased wool production compared with the control, and dietary addition of 0.4% to 0.6% Met in LP increased wool production to the control level. These results indicate that Met is an important nutrient for hair formation and growth. Met reversed the LP feeding-induced low coarse wool rate and wool fineness, which may be related to the increased primary follicle density. The primary follicle can develop into coarse hair with a cavum medullary. Dietary addition of 0.4% to 0.6% Met significantly increased the primary follicle density compared to the LP group, indicating that Met could stimulate primary follicle development.

### IGF1 signaling was associated with the Met-regulated hair follicle development

Hair follicles are found throughout the epidermis and dermis of mammalian skin, and fine regulation of the signalling network between hair follicle stem cells in the epidermis and hair papilla cells in the dermis is critical for initiating hair follicle regeneration [15]. *IGF1* could stimulate the development of hair follicles by regulating the proliferation of keratinocytes to maintain the extension of hair follicles during the growth period and postpone the retrogressive phase [16]. Previous studies showed that the lack of either protein or Met in diet suppressed *IGF1* content in mouse serum, while the dietary addition of Met significantly increased *IGF1* expression in liver of rabbits [17]. In our study, feeding LP downregulated *IGF1* expression in rabbit skin compared with the control, which was recovered fully by dietary addition of 0.4% Met. These results suggest that *IGF1* is an important target at the dietary protein level of Met regulating hair follicle development.

### *KAP* gene was associated with the Met-regulated hair follicle development

In mammals, hair is primarily composed of keratins, which account for 65% to 95% of total hair fibres and constitute the framework of the hair fibre itself [17]. *KAP* genes are expressed largely in the whole periodic cycle of hair follicle development [6]. Keratin has many disulfide bonds made of cysteine. Met can be metabolized into cysteine and cystine to provide an adequate substrate for keratin protein formation and affect hair growth. In the previous study, *KAP3.1* and *6.1* gene expression was higher in rabbits with high wool density than in rabbits with low wool density [18]. LP treatment reduced the expression of *KAP3.1* and *KAP6.1*, and the addition of Met significantly increased the expression of *KAP3.1* and *KAP6.1*, indicating that *KAP3.1* and *KAP6.1* are the key targets involved in Met promoting hair growth in LP-fed rabbits.

### Versican signaling was involved in the Met-regulated hair follicle development

Versican is a multifunctional proteoglycan involved in the induction of hair morphogenesis, initiation of hair regeneration, and maintenance of hair growth [19]. In our present study, LP feeding did not significantly affect versican gene expression in rabbit skin compared with the control, indicating that versican may not be associated with LP-inhibited hair follicle development. However, versican mRNA levels in the LP+Met group increased 5-fold compared with the control and LP groups, indicating that rabbit versican gene expression is sensitive to dietary Met levels. In addition, versican may be involved in the process by which Met regulates hair follicle development.

### Wnt10b, EGF, TGFβ, Noggin, and HGF signaling were not associated with the Met-regulated hair follicle development

Wnt signalling pathway could stimulate early hair follicle development and cyclic regeneration. Vitro studies showed that *Wnt10b* promotes the differentiation of skin epithelial cells into the hair shaft and induces the transition from the resting to anagen phase of hair follicles in mice [20]. In our study, low protein levels significantly inhibited *Wnt10b* mRNA level, indicating that rabbit skin *Wnt10b* signalling pathway is affected by dietary protein levels. In addition, the addition of Met to LP did not alleviate the inhibitory effect of LP on *Wnt10b* expression, suggesting that *Wnt10b* may not a key target of Met improving hair follicle development.

Studies have shown that appropriate concentrations of *EGF* can promote the proliferation of hair follicle stem cells and hair papilla cells and alter the cell cycle [21]. Noggin is expressed in the dermal papilla and secreted into adjacent hair follicle epithelial cells, which activates the proliferation of hair follicle epithelial cells and improves hair follicle growth [22]. In contrast, *TGFβ* is a member of the transforming growth factor superfamily and plays a negative role in the regulation of periodic hair follicle regeneration [23]. In our study, LP treatment or Met supplementation had no significant effect on the expression of *EGF*, *TGFβ1*, *TGFβ2*, and Noggin, indicating that these genes may not be the major targets of dietary protein levels or Met regulating hair follicle development.

*HGF* has been reported to promote follicular growth and enhance DNA synthesis [24]. Information about the effect of Met on *HGF* gene expression is scarce. In our study, LP treatment had no significant change in versican expression, while the addition of Met decreased *HGF* expression. The mechanism of Met regulating *HGF* gene expression is uncertain and needs further study.

### Dietary addition of Met improves nitrogen utilization efficiency in Angora rabbits fed a low-protein diet

The digestive metabolism of nitrogen reflects the AA balance and the efficiency of protein deposition in the diet. Proper protein levels and AA ratios can optimize protein utilization efficiency, improve apparent nitrogen digestibility, and reduce nitrogen excretion. The nitrogen intake and urinary nitrogen of mink gradually decreased with the reduction of dietary protein levels. Reducing the dietary crude protein content from 17.7% to 16.2% can reduce nitrogen excretion [25]. Reducing dietary protein levels from 20% to 12% and supplementing with essential AAs resulted in lower urinary nitrogen and total nitrogen excretion in pigs [26]. These results are consistent with our findings that feeding LP significantly reduced nitrogen intake and excretion in Angora rabbits, especially urinary nitrogen. An insufficient supply of limiting AAs affects the utilization of other AAs, resulting in the excretion of unused nitrogen in urine. Met has been reported to be the first limiting AA for fur animals [27]. Met supplementation significantly increased the digestibility of dry matter and crude protein in rabbits. The dietary addition of 0.35% methionine significantly increased the protein digestibility of growing rabbits [28]. The addition of Met in diet has a favorable effect on nitrogen balance, nitrogen retention coefficient, growth performances and feed efficiency of rabbits [29]. Similar results were obtained in our study, indicating that the addition of 0.4% Met to LP significantly reduced faecal and urinary nitrogen and improved nitrogen utilization efficiency and biological potency in Angora rabbits. These results suggest that Met can improve the AA balance of LP-fed Angora rabbits, increase protein utilization, reduce nitrogen emissions to the environment, and increase feed conversion efficiency.

## CONCLUSION

The addition of Met in LP could improve wool production performance and promote hair follicle development, which may be associated with *IGF1*, *KAP* and the versican signaling. In addition, dietary addition of Met could increase feed efficiency and reduce nitrogen emissions in LP-fed Angora rabbits.

## Figures and Tables

**Table 1:** Composition and nutrient levels of basal diets (air-dry basis)

Item	Treatment^1)^				

	Control	LP	LP+0.2%Met	LP+0.4%Met	LP+0.6%Met
Feed ingredient (%)					
Corn	15.00	17.00	17.00	17.00	17.00
Soybean meal	18.00	13.00	13.00	13.00	13.00
Wheat bran	20.00	20.00	20.00	20.00	20.00
Peanut vine	30.00	33.00	33.00	33.00	33.00
Soya bean stem meal	10.00	10.00	10.00	10.00	10.00
Artemisia argyi	5.00	5.00	5.00	5.00	5.00
Bean oil	0.80	0.80	0.70	0.50	0.30
Methionine	-	-	0.10	0.30	0.50
Premix^2)^	1.10	1.10	1.10	1.10	1.10
Total	100	100	100	100	100
Nutrient composition (%)					
DE (MJ/kg)^3)^	10.83	10.97	10.97	10.97	10.97
CP (%)	17.09	15.16	15.13	15.15	15.18
EE (%)	3.53	3.08	3.29	3.22	2.98
Ash (%)	9.06	8.79	8.39	8.59	8.59
CF (%)	15.10	15.22	15.85	15.74	16.13
ADF (%)	18.82	21.52	20.50	19.50	20.68
NDF (%)	32.24	32.76	31.36	31.67	33.19
ADL (%)	3.20	3.67	3.59	3.00	3.34
Ca (%)	0.99	1.04	1.11	0.99	1.05
P (%)	0.64	0.63	0.64	0.63	0.60
Met (%)	0.33	0.31	0.42	0.62	0.81

DE, digestible energy; CP, crude protein; EE, ether extract; Ash, Ash content; CF, crude fibre; ADF, aerage daily feed intake; NDF, neutral detergent fiber; ADL, acid detergent lignin; Ca, calcium; P, phosphorus; Met, methionine.

1) Control, normal diet; LP, low protein diet; LP+0.2% Met, LP+0.2% methionine; LP+0.4% Met, LP+0.4% methionine; LP+0.6% Met, LP+0.6% methionine.

2) The premix provided the following per kg of diets: Vit A 8,000 IU, Vit D_3_ 400 IU, Vit E 50 mg, Vit K 30.8 mg, Vit B_1_ 0.36 mg, Vit B_2_ 0.4 mg, Vit B_6_ 0.6 mg, Vit B_12_ 0.006 mg, Nicotinic acid 4 mg, Folate 0.12 mg, Calcium Pantothenate 7 mg, Biotin 0.04 mg, Fe (as ferrous sulfate) 50 mg, Mn (as manganese sulfate) 4 mg, Cu (as copper sulfate) 20 mg, Zn (as zinc sulfate) 50 mg, Choline chloride 1,000 mg, Iimestone 10,000 mg, Nacl 3,000 mg, Lys 2,800 mg.

3) Digestible energy was calculated, while the other nutrient values were measured.

**Table 2:** Gene-specific primer sequences used for gene transcription analyses

Gene	Genebank accession number	Primers sequences (5′→3′)	Product size (bp)
*GAPDH*	NM_001082253.1	F: TCACCATCTTCCAGGAGCGA R: CACAATGCCGAAGTGGTCGT	293
*IGF1*	NM_001082026.1	F: GTGGAGACAGGGGCTTTTATTT R: TGTTGGTAGATGGAGGCTGATA	228
*Wnt10b*	NM_002711076	F: TGTGCCATCCCTCTTCCTTA R: GGCTCCACCTCTAACTTCTGC	150
*Noggin*	XM_002719279	F: CCAGCACTACCTCCACATCC R: GCGTCTCGTTCAGATCCTTC	123
*Versican*	XM_017344567	F: AGGTCAGCCCTCTCAAGACA R: TCTGTTCTTCCCGAGTGGTC	119
*HGF*	NM_001168707	F: TTGTCCTCTTGCTCGTTGTG R: GTTCGTGTTGGAATCCCATT	120
*EGF*	XM_008267485.1	F: AATGCCAACTGCACAAACAC R: CTGAAATGGCGGAACAGAAT	102
*KAP3.1*	HM147283	F: ACCTCTGACAAATGCTGCC R: CCAGCAGGATGAGACATAGATT	138
*KAP6.1*	M95718.1	F: GCCAATGAAAGAAGCACC R: GTTGAGGTTGTCCTTGGG	131
*TGFβ1*	NM_008249704.1	F: CCGTTTCTTTCGTGGGATAC R: GGTAAGGGAGGAGGGTCTCA	108
*TGFβ2*	NM_001082660.1	F: GAGAGGAGCGACGAGGAGTA R: TGAGCCAGAGGGTGTTGTAA	108

*GAPDH*, glyceraldehyde 3-phosphate dehydrogenase; *IGF1*, insulin-like growth factor 1; *Wnt10b*, Wingless-type MMTV integration site family, member 10B; *HGF*, hepatocyte growth factor; *EGF*, epidermal growth factor; *KAP3.1*, Keratin Association Protein 3.1; *KAP6.1*, Keratin Association Protein 6.1; *TGFβ1*, transforming growth factor β1; *TGFβ2*, transforming growth factor β2.

**Table 3:** Effect of a low-protein diet and a low-protein diet supplemented with different levels of methionine on wool production and quality in Angora rabbits

Items	Treatments^1)^					p-value

	Control	LP	LP+0.2% Met	LP+0.4% Met	LP+0.6% Met	
Wool production (n = 5)						
Wool yield (g)	294.58±16.69^a^	260.21±11.42^b^	263.81±8.42^b^	319.35±22.73^a^	312.11±13.52^a^	0.038
Total feed intake (kg)	12.08±0.01^b^	12.14±0.02^ab^	12.48±0.02^a^	12.52±0.01^a^	12.21±0.02^ab^	0.046
FCR	41.35±1.98^ab^	46.95±1.70^a^	47.52±1.63^a^	39.99±2.78^b^	39.43±1.73^b^	0.020
Wool quality (n = 10)						
Wool length (cm)	5.54±0.14	5.40±0.16	5.37±0.11	5.57±0.17	5.66±0.16	0.579
Coarse wool rate (%)	9.78±3.24^a^	2.53±0.64^b^	2.21±0.41^b^	7.60±1.54^ab^	12.40±2.18^a^	0.001
Wool fineness (μm)	16.48±0.98^a^	14.65±0.23^b^	14.82±0.85^b^	16.32±0.61^a^	18.15±0.68^a^	0.008

Data are presented as means±standard error.

FCR, feed conversion ratio.

1) Control, normal diet; LP, low protein diet; LP+0.2%Met, LP+0.2% methionine; LP+0.4%Met, LP+0.4% methionine; LP+0.6%Met, LP+0.6% methionine.

a,b Values within a row with different superscripts differ significantly (p<0.05).

**Table 4:** Effect of a low-protein diet and a low-protein diet supplemented with different levels of methionine on hair follicle density in Angora rabbits (n = 10)

Items	Treatments^1)^					p-value

	Control	LP	LP+0.2% Met	LP+0.4% Met	LP+0.6% Met	
Total follicle density (n/mm^2^)	135.73±9.30	127.83±8.04	143.63±6.15	140.83±6.36	132.89±6.72	0.578
Primary follicle density (n/mm^2^)	7.20±0.59^ab^	6.00±0.63^c^	5.41±0.41^c^	6.58±0.37^bc^	8.52±0.45^a^	<0.001
Secondary follicle density (n/mm^2^)	128.83±9.34	121.83±7.97	138.22±6.13	134.26±6.35	124.37±6.59	0.500

Data are presented as means±standard error.

1) Control, normal diet; LP, low protein diet; LP+0.2% Met, LP+0.2% methionine; LP+0.4% Met, LP+0.4% methionine; LP + 0.6%Met, LP+0.6% methionine.

a–c Values within a row with different superscripts differ significantly (p<0.05).

**Table 5:** Effect of a low-protein diet and a low-protein diet supplemented with different levels of methionine on nitrogen metabolism in Angora rabbits (n = 10)

Items	Treatments^1)^					p-value

	Control	LP	LP +0.2% Met	LP +0.4% Met	LP+0.6% Met	
Intake nitrogen (g/d)	4.16±0.15^a^	3.74±0.13^b^	3.66±0.06^b^	3.64±0.10^b^	3.69±0.07^b^	0.011
Faecal nitrogen (g/d)	1.09±0.07^a^	0.87±0.03^b^	0.84±0.03^b^	0.75±0.06^b^	0.76±0.06^b^	<0.001
Urinary nitrogen (g/d)	1.55±0.03^a^	1.31±0.04^b^	1.27±0.03^b^	1.32±0.02^b^	1.32±0.02^b^	<0.001
Nitrogen retention (d/g)	1.53±0.10	1.56±0.11	1.56±0.06	1.58±0.13	1.62±0.09	0.974
Apparent nitrogen digestibility (%)	73.88±0.97^b^	76.60±1.02^ab^	77.16±0.95^a^	79.30±1.96^a^	79.50±1.65^a^	0.049
Nitrogen utilization rate (%)	36.47±1.30^b^	41.42±1.71^ab^	42.40±1.17^a^	43.08±2.43^a^	43.77±1.84^a^	0.049
Nitrogen biological value (%)	49.39±1.78^b^	53.99±1.59^ab^	54.94±1.23^a^	54.11±1.81^a^	54.96±1.34^a^	0.047

Data are presented as means±standard error.

1) Control, normal diet; LP, low protein diet; LP+0.2% Met, LP+0.2% methionine; LP+0.4% Met, LP+0.4% methionine; LP+0.6% Met, LP+0.6% methionine.

a,b Values within a row with different superscripts differ significantly (p<0.05).
