# Supplementary material for: Dietary methionine supplementation to a low-protein diet improved hair follicle development of Angora rabbits
Source: Anim Biosci. 2022 Nov 14;36(6):920–8. doi: 10.5713/ab.22.0286 (PMC10164532; doi:10.5713/ab.22.0286)
Supplement: Supplementary file 1 [file ab-22-0286-Supplementary-1.pdf]

Dietary methionine supplementation improved hair follicle development in low-protein diet-fed Angora rabbits

Man *Zhao*[1], Tongtong *Wang*[1], Bin *Wang*[2], Chuanhua *Liu*[2], Fuchang *Li*[1*] and Lei *Liu*[1*]

Supplementary Material S1 The detailed material for the determination of the chemical composition of the diets and nitrogen metabolism

Crude protein was determined by automatic Kjeldahl nitrogen meter (Foss, Denmark); crude fat EE was determined by Soxhlet extraction method; crude fiber, neutral detergent fiber and acid detergent fiber were determined by van's detergent fiber analysis; ash was determined by ashing method; calcium was determined by potassium permanganate titration; phosphorus was determined by molybdenum yellow colorimetry (Van Soest and Moore, 1965).

According to the experimental data, the apparent digestibility is calculated as follows: apparent digestibility of certain nutrients in diet (%) = (content of certain nutrients eaten-content of corresponding nutrients in feces) / content of certain nutrients eaten × 100%

After all the samples were collected, the faeces and urine samples from each rabbit were mixed. The faecal samples were dried in a drying box at 65 °C for 72 hours. After drying, each sample was opened and weighed twice after 24 hours of moisture recovery. The sample was crushed by a grinder and sealed at room temperature. The urine

samples were treated with 10%H_2SO_4 solution to measure nitrogen fixation (Liu et al., 2021).

The nitrogen contents of the feed, faeces, and urine samples were determined using a fully automated Kjeltec 8400 (Foss, Denmark) nitrogen tester. The equations for each nitrogen balance indicator were calculated as follows:

Digestible nitrogen (DN) (g/d) = Intake nitrogen (IN)-Faecal excreted nitrogen (FN)

Retention nitrogen (RN) (g/d) = Intake nitrogen (IN)-Faecal nitrogen (FN)-Urine nitrogen (UN)

Nitrogen apparent digestibility (NAD) (DN/IN, %) = Digestible energy (DN)/Intake nitrogen (IN)x100%

Nitrogen utilization rate (NUR) (RN/IN, %) = Retention nitrogen (RN)/Intake nitrogen (IN)x100%

Nitrogen biological value (NBV) (RN/DN, %) = Retention nitrogen (RN)/Digestible energy (DN)x100%

Van Soest PJ and Moore LA 1965. New chemical methods for analysis of forages for the purpose of predicting nutritive value. Proctect of the IX International Grassland Congress, 783-789.

Liu M, Li C, Tang H, Gong M, Yue Z, Zhao M, Liu L and Li F 2021. Dietary lysine supplementation improves growth performance and skeletal muscle development in rabbits fed a low protein diet. Journal of Animal Physiology and Animal Nutrition.
