# Supplementary material for: Dietary methionine supplementation to a low-protein diet improved hair follicle development of Angora rabbits
Source: Anim Biosci. 2022 Nov 14;36(6):920–8. doi: 10.5713/ab.22.0286 (PMC10164532; doi:10.5713/ab.22.0286)
Supplement: Supplementary file 2 [file ab-22-0286-Supplementary-2.pdf]

Supplementary Material S2 Detailed materials for paraffin sectioning and staining

The fixed skin samples were dehydrated in a series of graded ethanol solutions (50%, 70%, 80%, 85%, 90%, 95%, and anhydrous ethanol), and then the samples were embedded in paraffin. When each skin sample was crosscut, the surface of the epidermis was at the top of the paraffin section and was as flat as possible to ensure that the surface of the epidermis was parallel to the section of the paraffin knife. Using an automatic slicer, the first set of sections with 5 μm thick serial sections parallel to the surface of the skin was collected until there were 5-10 primary follicles with sebaceous glands. The second and third groups of slices were collected at intervals of 50 μm in each group, and the sections were cut into three different depths of the sebaceous glands to avoid the omission of secondary follicles. The tissue sections were stained with haematoxylin and 0.1% eosin dye (Sigma–Aldrich, St. Louis, MO). After staining, the sections were dehydrated in 70%, 80%, 90%, and 100% ethanol solutions. After drying, the slides were sealed with neutral resin and coverslips, and the sealed tissue sections were dried in an electric oven at 37 °C. The number of hair follicles was counted using an Olympus CX-41 phase-contrast microscope (Olympus, Tokyo, Japan). For each sample, each slice was randomly photographed under the same magnification (100 ×) from 3 visual fields, and the average hair follicle density was calculated. The hair follicles of each counting area plus all the hair follicles that intercepted the top and left edges of each counting area were counted; hair follicles that intercepted the bottom and right margins of the counting areas were not included in the counts. There are two types of hair follicles in the skin of Rex rabbits: primary follicles and secondary follicles,

which are easy to distinguish according to their specific characteristics. In short, the size of primary follicles is larger than that of secondary follicles. Primary follicles also contain large fibres, a narrow skin layer, and a very large medulla cross-section, while secondary follicles contain only a small medulla or no medulla. Secondary follicles surround the primary follicles on the opposite side in each follicle group. In addition, primary follicles are accompanied by accessory structures such as a sebaceous gland and an arrector pili muscle.
