# Supplementary material for: Dietary methionine supplementation to a low-protein diet improved hair follicle development of Angora rabbits
Source: Anim Biosci. 2022 Nov 14;36(6):920–8. doi: 10.5713/ab.22.0286 (PMC10164532; doi:10.5713/ab.22.0286)
Supplement: Supplementary file 3 [file ab-22-0286-Supplementary-3.pdf]

Supplementary Material S3 Detailed materials for total RNA extraction and real-time quantitative PCR

Quantitative real-time PCR (RT–PCR) labelled by SYBR Green I was used to quantify the gene expression in hair follicles. Total RNA was isolated and extracted by using the guanidine isothiocyanate method with TRIzol reagents (Invitrogen, San Diego, CA, USA). The quality of RNA after DNase treatment was tested by electrophoresis on an agarose gel, and the quantity of RNA was determined using a biophotometer (Eppendorf, Germany). RT-PCRs (10 μL) consisted of 500 ng total RNA, 5 mmol/L MgCl2, 1 μL RT buffer, 1 mmol/L dNTPs, 2.5 U AMV, 0.7 nmol/L oligo d(T), and 10 U ribonuclease inhibitor (TaKaRa Biotechnology, Co., Ltd. Dalian, P. R. China). Real-time PCR analysis was conducted using the Applied Biosystems 7500 Real-time PCR System (Applied Biosystems, Foster, CA, USA). Each RT-reaction served as a template in a 20 μL PCR containing 0.2 μmol/L of each primer and SYBR green master mix (Takara Biotechnology, Co., Ltd. Dalian, P. R. China). The primer-set sequences are described in Table 1. Real-time PCRs were performed at 95 °C for 10 s, followed by 40 cycles at 95 °C for 5 s, and at 60 °C for 40 s. SYBR green fluorescence was detected at the end of each cycle to monitor the amount of PCR product. A standard curve was plotted to calculate the efficiency of the real-time PCR primers. The PCR data were analysed with the $2^{-\Delta\Delta}$CT method. The mRNA levels of the target genes were normalized to the level of glyceraldehyde 3-phosphate dehydrogenase (*GAPDH*). Based on the cycle threshold (CT) values, *GAPDH* mRNA expression was stable across treatments in this study (P>0.1). The ΔCT was calibrated against the average of the

control rabbits. The linear amount of target molecules relative to the calibrator was calculated by $2^{-\Delta\Delta}$CT. Therefore, all gene transcription results are reported as the n-fold difference relative to the calibrator. The specificity of the amplification product was verified.
