# Supplementary material for: Dietary methionine supplementation to a low-protein diet improved hair follicle development of Angora rabbits
Source: Anim Biosci. 2022 Nov 14;36(6):920–8. doi: 10.5713/ab.22.0286 (PMC10164532; doi:10.5713/ab.22.0286)
Supplement: Supplementary file 4 [file ab-22-0286-Supplementary-Fig-1.pdf]

Supplementary Figure S1 The reference gene (GADPH) in different groups of rabbit hair follicles

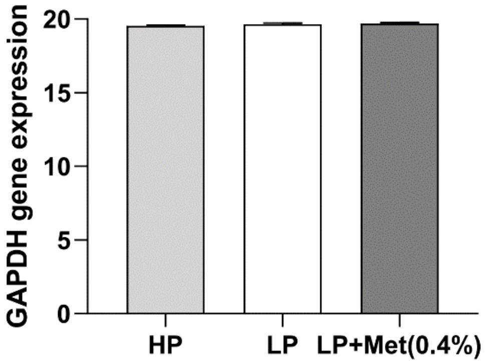

Abbreviations: GAPDH = glyceraldehyde 3-phosphate dehydrogenase.
